# MicroRNA Expression in Macrophages Regulates Antibody-Dependent Effector Function and B-cell Lymphoma Therapy

**DOI:** 10.1158/2767-9764.CRC-25-0485

**Published:** 2026-11-09

**Authors:** Jemma Longley, Lekh N. Dahal, Russell B. Foxall, Stephen Thirdborough, Margaret Ashton-Key, Matthew J. Carter, Rosanna C. Smith, Robert Kemp, Peter W.M. Johnson, Andrew Davies, Stephen A. Beers, Mark S. Cragg

**Affiliations:** 1 Antibody and Vaccine Group, Centre for Cancer Immunology, School of Cancer Sciences, Faculty of Medicine, Southampton General Hospital, University of Southampton, Southampton, United Kingdom.; 2 Department of Pharmacology and Therapeutics, Institute of Systems, Molecular and Integrative Biology (ISMIB), University of Liverpool, Liverpool, United Kingdom.; 3 Department of Cellular Pathology, University Hospital Southampton, Southampton, United Kingdom.; 4 Clinical Trials Unit, Centre for Cancer Immunology, School of Cancer Sciences, Faculty of Medicine, Southampton General Hospital, University of Southampton, Southampton, United Kingdom.; 5 Centre for Cancer Immunology, School of Cancer Sciences, Faculty of Medicine, Southampton General Hospital, Southampton, United Kingdom.; 6 Institute for Life Sciences, University of Southampton, Southampton, United Kingdom.

## Abstract

CD163^+^ tumor-associated macrophages (TAM) are associated with poor response to immunochemotherapy in patients with diffuse large B-cell lymphoma and comprise a significant proportion of the tumor microenvironment (TME). Targeting these TAMs to reverse immunosuppression is an attractive approach for improving treatment response rates. A change in microRNA (miRNA) expression has the potential to modify hundreds of genes by interacting with multiple messenger RNA (mRNA) targets, thereby altering the wider cellular transcriptional profile. The manipulation of miRNAs that control macrophage behavior may switch the global transcriptomic profiles of immunosuppressive macrophages to more activated, antitumor states, resulting in the reversal of immune resistance and enhancement of monoclonal antibody (mAb) therapy. Our studies show a significant upregulation of miR-142-5p in immunosuppressive macrophages stimulated with IL4/IL13 compared with lipopolysaccharide (LPS)/IFNγ-stimulated macrophages and nonpolarized controls. This upregulation of miR-142-5p in macrophages stimulated with immunoinhibitory cytokines IL10 and TGFβ was also demonstrated. Inhibition of miR-142-5p expression in IL4/IL13-stimulated macrophages led to a significant increase in FcγRIIIA, CD40, and CD47 expression with enhanced phagocytosis of chronic lymphocytic leukemia (CLL) cells opsonized with rituximab when compared with nontransfected controls. Expression of miR-142-5p in diagnostic samples of patients with diffuse large B-cell lymphoma (DLBCL) was assessed using miRScope assays. These were combined with immunohistochemistry (IHC) staining of CD206 to identify immunosuppressive macrophages. Combination of high miR-142-5p and CD206 expression correlated with a reduction in progression-free survival (PFS) following standard rituximab containing chemotherapy regimens in 38 patients. Together, these studies indicate the potential of targeting miRNA to repolarize macrophages and improve treatment success.

**Significance:**

The manipulation of macrophage polarization within the TME has the potential to enhance immunotherapies used across many different cancer types. The use of miRNA inhibitors to target the immune system in a cell-specific way using antibody oligonucleotide conjugates has the potential to reduce toxicity historically associated with miRNA therapeutics and change the phenotype of cells at the transcriptomic level.

## Introduction

Tumor-associated macrophages (TAM) are thought to downregulate cytotoxic T-cell responses, promote angiogenesis and tumor invasion, and enhance tumor progression (1). However, the role of TAM infiltration in diffuse large B-cell lymphoma (DLBCL) remains controversial. Some studies suggest that TAMs, identified using the marker CD68, predict a favorable prognosis (2). Conversely, high CD163 expression and a high CD163:CD68 ratio correlate with inferior survival outcomes in patients receiving rituximab, cyclophosphamide, doxorubicin, vincristine, and prednisolone (RCHOP) treatment (3, 4). This conflicting role of TAMs in DLBCL is likely affected by both the nature of IHC markers used and different treatment strategies implemented across the disease spectrum.

An additional marker, the C-type lectin mannose scavenger receptor, CD206, is primarily expressed on macrophages and endothelial cells and plays an important role in tissue homeostasis by removing mannoglycoproteins (5). High CD206 expression on TAMs has been linked to increased invasion and metastasis in ovarian and oral squamous cell cancers, leading to poor survival outcomes (6). However, other studies have associated CD206 expression with a pro-inflammatory phenotype. This conflicting role of CD206 in solid tumors highlights our inability to define macrophage function based on a single marker. This complexity is influenced by the immunologic microenvironment, tumor type, and type of anticancer treatment given (7).

There is growing recognition of potential phenotypic discordance between tissue-resident macrophages (TRM) and blood-derived macrophages as tissue-resident populations are developmentally imprinted by their local niche and maintain specialized transcriptional, metabolic, and functional programs (8). TRMs are independently established during embryonic development and are first seen in early gestation (9). Different subsets of TRMs have been identified depending on tissue type, including Kupffer cells, microglia, and alveolar macrophages residing in the liver, nervous system, and lung, respectively, which regenerate independently of monocyte recruitment during the steady state (8). Consequently, *in vitro* models using blood-derived macrophages can be limited in their ability to recapitulate tissue-specific immune behavior, stromal interactions, and disease-relevant responses seen *in vivo*. In a previous work, we have shown that the syngeneic BCL_1_ mouse lymphoma model induced a profound immunosuppression of TAMs, as indicated by modulation of their Fcγ receptor (FcγR) profile, leading to reduced efficacy of mAb immunotherapy (10). Notably, STING agonists reversed this, converting immunosuppressed TAMs to a pro-inflammatory phenotype, and improved animal survival when administered alongside standard anti-CD20 monoclonal antibody (mAb) immunotherapy (10). Understanding the mechanisms driving the suppressive TAM program may provide opportunities to exploit macrophage plasticity and reestablish a pro-inflammatory phenotype, enhancing phagocytosis, upregulating T-cell responses, and improving outcomes for patients with B-cell lymphoma.

Crucially, TAMs can reprogram their transcriptional profiles and switch between different polarization states in response to their microenvironment (10, 11) and stimuli. MicroRNAs (miRNA) have the potential to modify hundreds of genes by interacting with multiple messenger RNA (mRNA) targets, thereby altering the wider transcriptional profile and activity of cells (12). In this study, we focused on identifying and manipulating miRNAs in TAMs to enhance antibody-dependent phagocytosis in B-cell lymphoma.

## Materials and Methods

### Clinical samples and ethical approval

Human peripheral blood mononuclear cells (PBMC) from leukocyte cones were obtained from the University of Southampton Hospital Blood Donor Centre following ethical approval from the East of Scotland Research Ethics Service and were a by-product from healthy blood donors attending for platelet donations. Chronic lymphocytic leukemia (CLL) samples were obtained from Human Tissue Authority Licensed University of Southampton, School of Cancer Sciences Tissue Bank [research ethics committee (REC) number: 10/H0504/32], and used as cell targets in antibody-dependent cellular phagocytosis (ADCP) assays. Ethical approval for the use of tissue microarrays (TMA) was obtained by Southampton University Hospitals NHS Trust from Southampton and Southwest Hampshire Research Ethics Committee (REC number: 10/H0504/187). The use of primary human material from anonymized and consenting donors was reviewed and approved locally by the University of Southampton Faculty of Medicine Ethics Committee (19660.A11) and at a national level by the National Health Service (NHS)/Health and Social Care Research Ethics Committee (REC; IRAS: 186605).

Patient studies were conducted in accordance with the Declaration of Helsinki with written informed consent obtained from study participants. Survival data were extracted from electronic clinical records and stored at the University of Southampton NHS Trust IT systems. All data were anonymized and assigned a study number. Progression-free survival (PFS) was defined as the time from the first diagnosis to evidence of relapse, either by clinical judgment and/or imaging with computerized tomography (CT). Stage, age, and gender were also recorded to adjust for other variables in the analysis. RNA sequencing (RNA-seq) data available from The Cancer Genome Atlas (TCGA) was utilized from 48 DLBCL samples that were publicly available (data generated by TCGA Research Network: https://www.cancer.gov/tcga).

### Generation and polarization of human monocyte-derived macrophages

PBMCs isolated from leukocyte cones were cultured for 7 days with macrophage colony stimulating factor (MCSF; 100 ng/mL) to produce human monocyte-derived macrophages (hMDM) and then polarized with various stimuli (13). Cells were either not treated (MCSF 100 ng/mL), obtained in-house, or treated with IL4 plus IL13 (both 10 ng/mL; Thermo Fisher Scientific), IL10 (100 ng/mL; Thermo Fisher Scientific), or TGFβ (20 ng/mL; Thermo Fisher Scientific) to generate suppressed macrophages or lipopolysaccharide (LPS; 50 ng/mL; Sigma-Aldrich) + IFNγ (2 ng/mL; Thermo Fisher Scientific), Pam3CSK (100 μg/mL; InvivoGen), R848 (1 ng/mL; InvivoGen), or IFNγ (2 ng/mL) to mimic immunostimulatory macrophages for 48 hours.

### Transfection of miRNA inhibitors

Anti-miRNA reagents (Invitrogen) and the Lipofectamine RNAiMAX reagent (Thermo Fisher Scientific) were used to inhibit miRNA expression. On day 6 of hMDM generation, hMDMs were transfected with either a negative control (NC; scrambled unlabeled or fluorescein amidite labeled) or a specific anti-miRNA sequence using a Lipofectamine RNAiMAX reagent protocol at the same time as polarization (14). Cells were acquired 24 and 48 hours later by flow cytometry and analyzed by *de novo* FCS Express software (RRID: SCR_016431).

### Analysis of miRNA expression

RNA was extracted from differentially treated hMDMs using the mirVana kit (Thermo Fisher Scientific). The synthesis of cDNA from 100 ng of RNA was performed for each individual miRNA using TaqMan miRNA assays and miRNA reverse transcription kit (Thermo Fisher Scientific) and then stored at −80°C. Quality and concentration were checked using NanoDrop (Thermo Fisher Scientific). Analysis and fold change (FC) were calculated using either RU44 or RU48 as a housekeeping gene. Changes in the control condition versus experimental condition were calculated by producing the ΔΔCt value and presenting as FC using the 2^−ΔΔCt^ method (Microsoft Excel, RRID: SCR_016137).

### Phenotypic analysis

Differentially treated hMDMs were assessed for phenotypic changes using flow cytometry. hMDMs were stained with anti–human CD40 [Alexa Fluor; (AF) 488 clone ChiLob 7/4], CD38 PerCp (clone HIT2, RRID: AB394184), DC-SIGN APC (clone 9E9A8, RRID: AB1134055), CD11c PE (clone 3.9, RRID: AB10596967), CD11b PE (clone ICRF44, RRID: AB396849), CD206 (AF)488 (clone 15-2, RRID: AB3703376), and CD163 PE (clone GHI/61, RRID: AB2033943; BD Biosciences). FcγR staining was as described previously (FcγRIIA/B kind gift from BioInvent; ref. 15). Samples were acquired on a FACSCalibur flow cytometer (BD Biosciences), and data were analyzed with *de novo* FCS Express (RRID: SCR_016431) and FlowJo (RRID: SCR_008520).

### Phagocytosis assay and calculation of the phagocytic index

ADCP was assessed using primary human CLL cells stained with carboxyfluorescein diacetate succinimidyl ester (CFSE) as targets. Different macrophage populations were used as effector cells polarized with stimuli 48 hours (as above) prior to assessing ADCP. Each sample was run on a FACSCalibur flow cytometer, and the percentage of double-positive macrophages (FcγRIIIA+ CFSE+) was calculated for each condition using FlowJo software. Analysis by flow cytometry was used to determine the extent of ADCP and calculate the phagocytic index (PI) for each condition to assess phagocytic efficacy in the presence of anti-CD20 mAb (rituximab) compared with the anti-HER2 mAb (Herceptin) isotype control. The PI was calculated by dividing the percentage of phagocytic (FcγRIIIA+ and CFSE+) macrophages seen in the test condition minus isotype control by the percentage of phagocytic macrophages seen in the unstimulated condition to calculate a ratio (10).

### Immunohistochemistry

Immunohistochemistry (IHC) was performed on formalin-fixed, paraffin-embedded (FFPE) DLBCL sections arranged into multiple TMAs and then stained using the BOND RX Fully Automated Research Stainer (Leica Microsystems, RRID: SCR_025548). Three cores per patient were randomly chosen and arranged in triplicate with a total of 135 cores per slide. Three cores from normal kidney tissue were included to orientate the slide. The study population included patients with DLBCL germinal B center and activated B cell subtypes, from any stage of disease who were treatment naïve. TMAs were deparaffinized, pretreated for heat-induced antigen retrieval (BOND ER2 protocol), and incubated with hydrogen peroxide followed by the CD206 primary antibody before incubation with 3,3′-diaminobenzidine (DAB) at varying dilutions (RRID: AB_571922). Lung adenocarcinoma sections were used as a positive control for CD206 expression.

### miRNAscope

The miRNAscope *in situ* single-plex hybridization red chromogenic probe plus the miRNAscope LS assay red reagent kit was used to detect miRNA expression (Bio-Techne ACD). This was performed in combination with IHC for CD206 using the BOND RX Fully Automated Research Stainer for DLBCL TMAs, with normal lymph node sections used as a control. A universal probe targeting nuclear U6 RNA, which is highly expressed across both mouse and human tissues, was used as a positive miRNA control and allowed assessment of RNA quality prior to sample inclusion in the analysis. A probe targeting a scrambled miRNA sequence with no known biological function was used as an NC and allowed assessment of any nonspecific staining. Specific miRNA sequence target probes were used to measure miRNA expression in different patient cores. Once staining was complete, slides were dipped into pure xylene and coverslips mounted using EcoMount (Bio-Techne ACD).

### Digital pathology analysis

Stained TMA slides were scanned using the ZEISS Imager Z1 upright microscope with an AxioCam MRc 5 camera (ZEISS), using a predefined scanning protocol (RRID: SCR_023747). Three regions of interest (ROI) from each core within each TMA were randomly selected and exported to ImageJ for analysis (RRID: SCR_003070). Scoring of miRNAs was performed as per the manual provided by the manufacturer. TMAs with high miRNA expression were defined as having an miRNA score of 3 to 4, and those with low miRNA expression defined as a score of 0 to 2. Each ROI was scored separately and an average score across all 3 ROIs assigned. CD206 expression in each TMA core was counted and quantified into high versus low groups defined by a cutoff >30 cells staining positive and <30 cells staining positive, respectively. All TMAs were reviewed by an experienced pathologist to check quality of staining prior to analysis.

### Statistical analysis

GraphPad Prism 6.0 was used for statistical analysis (RRID: SCR_002798). Data with normal distribution were expressed as mean + standard deviation for 3 or more independent donors. The one-way ANOVA test was used for comparison between two separate groups. For multiple groups, the two-way ANOVA test was used. A *P* value < 0.05 was considered significant at the 95% confidence interval. Asterisks denote statistical significance (*, *P* < 0.05; **, *P* < 0.01; ***, 0.001; ****, *P* < 0.0001).

## Results

### Identification of TAM-associated miRNAs in DLBCL

**Figure 1.**
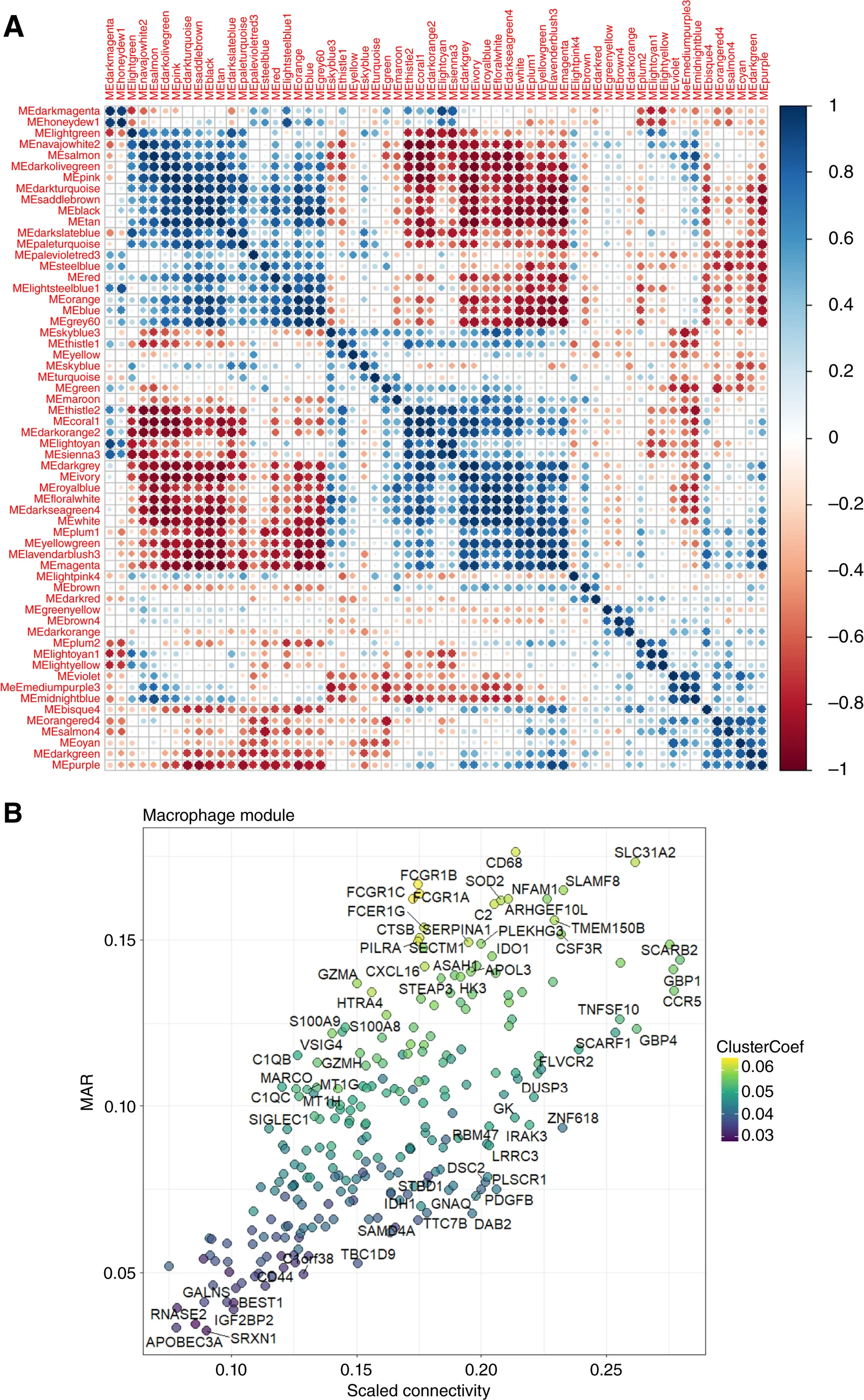
Bioinformatics analysis of miRNA expression in DLBCL and immunosuppressive macrophages within TCGA, miRBase, and TargetScan. **A,** Heatmap showing correlation of modules with each other, in which blue represents positive correlation and red represents negative correlation; 13,799 expressed genes summarized by 60 modules; the size and color of each circle indicates the strength of correlation between two modules using the Spearman correlation method. **B,** Network topology of the macrophage-enriched (cyan) co-expression module. Each point represents one gene within the cyan module. The *x*-axis shows scaled intramodular connectivity (normalized sum of adjacencies to other module genes; higher values denote hub genes). The *y*-axis shows maximum adjacency ratio (MAR; the fraction of a gene’s total connectivity contributed by its strongest single connection; higher MAR indicates dependence on a single partner). Points are colored according to the clustering coefficient (local neighborhood interconnectedness). The observed relationship between connectivity and MAR indicates that highly connected genes participate through distributed co-expression rather than single dominant edges, consistent with a robust macrophage transcriptional program.

To infer miRNAs associated with TAMs in DLBCL, we conducted a weighted gene co-expression network analysis using transcriptomic and matched miRNA data from the TCGA DLBCL cohort (*n* = 48) available from https://www.cancer.gov/tcga. This dataset included 13,779 actively expressed genes, which were grouped into 60 modules. We then performed cell-type enrichment analysis using the Enrichr platform to identify modules enriched with TAM-associated genes (16). Correlation analysis between modules revealed distinct clustering patterns, as visualized in Fig. 1A, in which blue indicates positive correlation and red indicates negative correlation. Figure 1B further illustrates the relationships among scaled connectivity, maximum adjacency ratio, and clustering coefficient for the top module enriched with macrophage-associated genes (PanglaoDB macrophages: adjusted *P* = 2.2e−36). To further investigate macrophage-associated gene networks, we identified gene modules enriched for CD163, a marker of immunosuppressive macrophages, using the GeneOverlap package in R. The gene expression matrix of the macrophage module was summarized by its first principal component, and the module eigengene values were correlated with miRNA expression data. The top 10 miRNAs showing the highest correlation with the macrophage module cyan were ranked (Supplementary Table S1). To further refine our analysis, we cross-referenced these miRNAs with publicly available databases, including miRBase and TargetScan, to identify conserved miRNAs with potential functional relevance in TAM biology (Supplementary Table S2; refs. 17, 18). This process identified additional miRNAs with known associations with immunosuppressive macrophage markers, which were prioritized for further investigation (Fig. 1D).

### Differential miRNA expression in polarized macrophages

**Figure 2.**
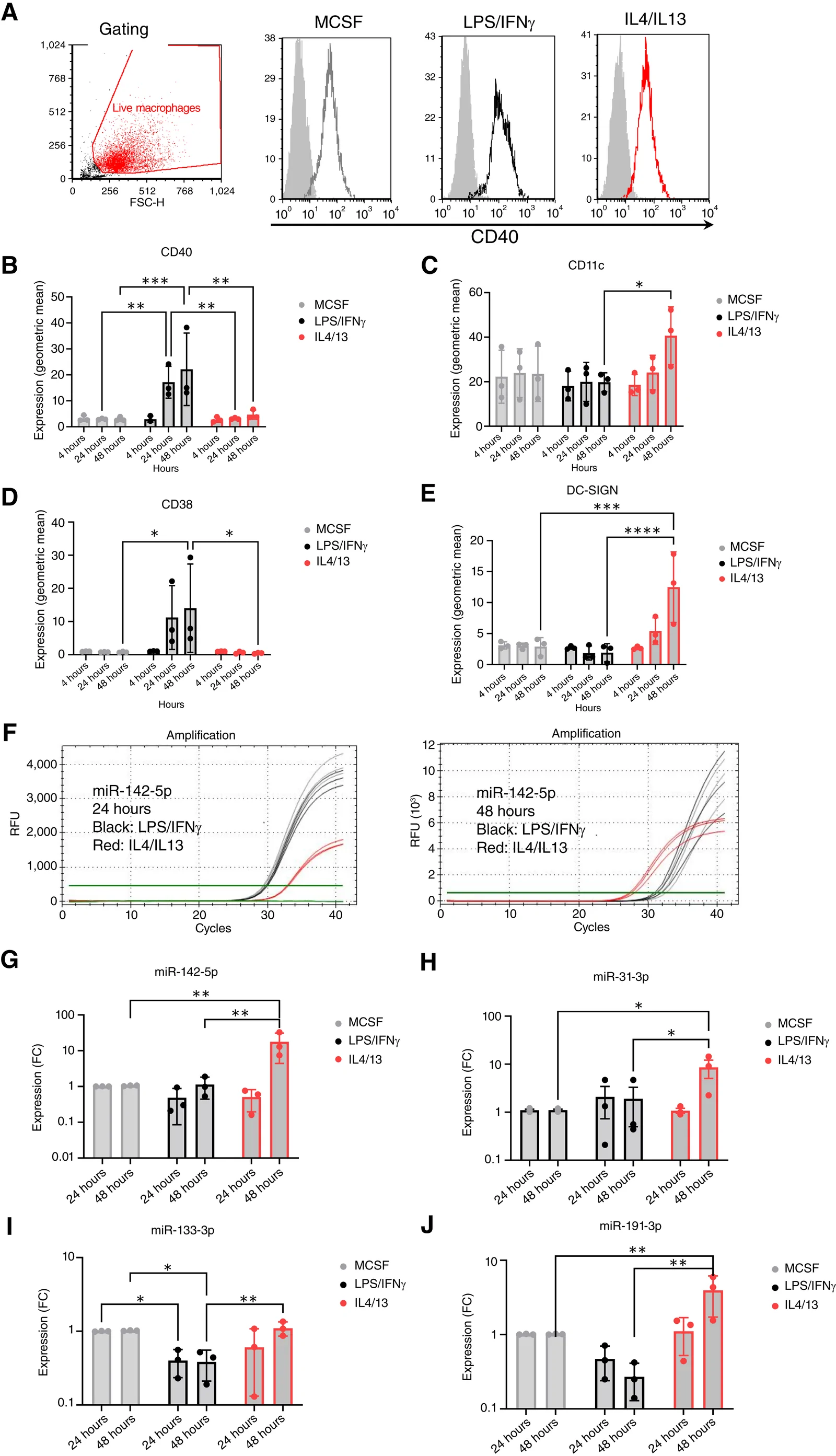
Comparative phenotype and miRNA expression in differentially polarized monocyte-derived macrophages. **A,** Representative FACS plots showing gating strategy and CD40 expression on differentially polarized hMDMs. CD40 (**B**), CD11c (**C**), CD38 (**D**), and DC-SIGN (**E**) expression on differentially polarized MDMs after 4, 24, and 48 hours of culture. Each graph depicts the mean ± SD expression of the respective molecules (geometric mean fluorescence intensity); each filled circle represents an individual donor. **F,** miR-142-5p qPCR cT curves for samples obtained 24 (left) and 48 hours (right) after polarization: LPS/IFNγ (black) and IL4/IL13 (red) macrophages performed in triplicate (*n* = 1). miR-142-5p (**G**), miR-31-3p (**H**), miR-133-3p (**I**), and miR-191-3p (**J**) expression in hMDMs after 24 and 48 hours of polarization. Data were normalized to RU44 and presented as FC relative to MCSF. Each graph shows the mean ± SD (*n* = 3 individual donors). Statistical analysis was carried out using a one-way ANOVA test; *, *P* < 0.05; **, *P* < 0.01; ***, *P* < 0.001; ****, *P* < 0.0001. FSC-H, forward scatter height; RFU, reletive fluroesence units.

To investigate the expression of miRNAs identified through bioinformatics analysis in different macrophage populations, we conducted *in vitro* polarization of hMDMs using immunostimulatory and immunoinhibitory polarizing reagents. Classical pro-inflammatory macrophages were generated by IFNγ/LPS stimulation, whereas immunosuppressive macrophages were induced using IL4/IL13, TGFβ, and IL10. These conditions were chosen to represent both the extremes of macrophage polarization (IL4/IL13 vs. LPS/IFNγ) and help reflect the immunosuppressive TME (IL10 and TGFβ). Flow cytometry analysis confirmed expected phenotypic changes, with CD40 and CD38 expression significantly upregulated in pro-inflammatory macrophages, whereas CD11c and DC-SIGN expression increased following IL4/IL13 treatment (Fig. 2A–E), in relation to nonpolarized macrophages.

Next, we examined the expression of candidate miRNAs identified in Fig. 1 across these macrophage populations. miR-142-5p, miR-31-3p, miR-191-3p, and miR-133-3p were significantly upregulated in IL4/IL13-polarized macrophages after 48 hours compared with both IFNγ/LPS-treated and nonpolarized MCSF macrophages (Fig. 2F–J). Among these, miR-142-5p demonstrated the highest FC difference between polarization states, aligning with prior studies highlighting its role in macrophage biology (19–21). This trend was replicated in macrophages stimulated with TGFβ and IL10, suggesting a role within the immunosuppressive TME (Supplementary Fig. S1). Based on these findings, we selected miR-142-5p for further investigation, employing a specific miRNA inhibitor to downregulate its expression and assess its impact on macrophage phenotype and function.

### miR-142-5p knockdown alters macrophage phenotype and enhances activation

**Figure 3.**
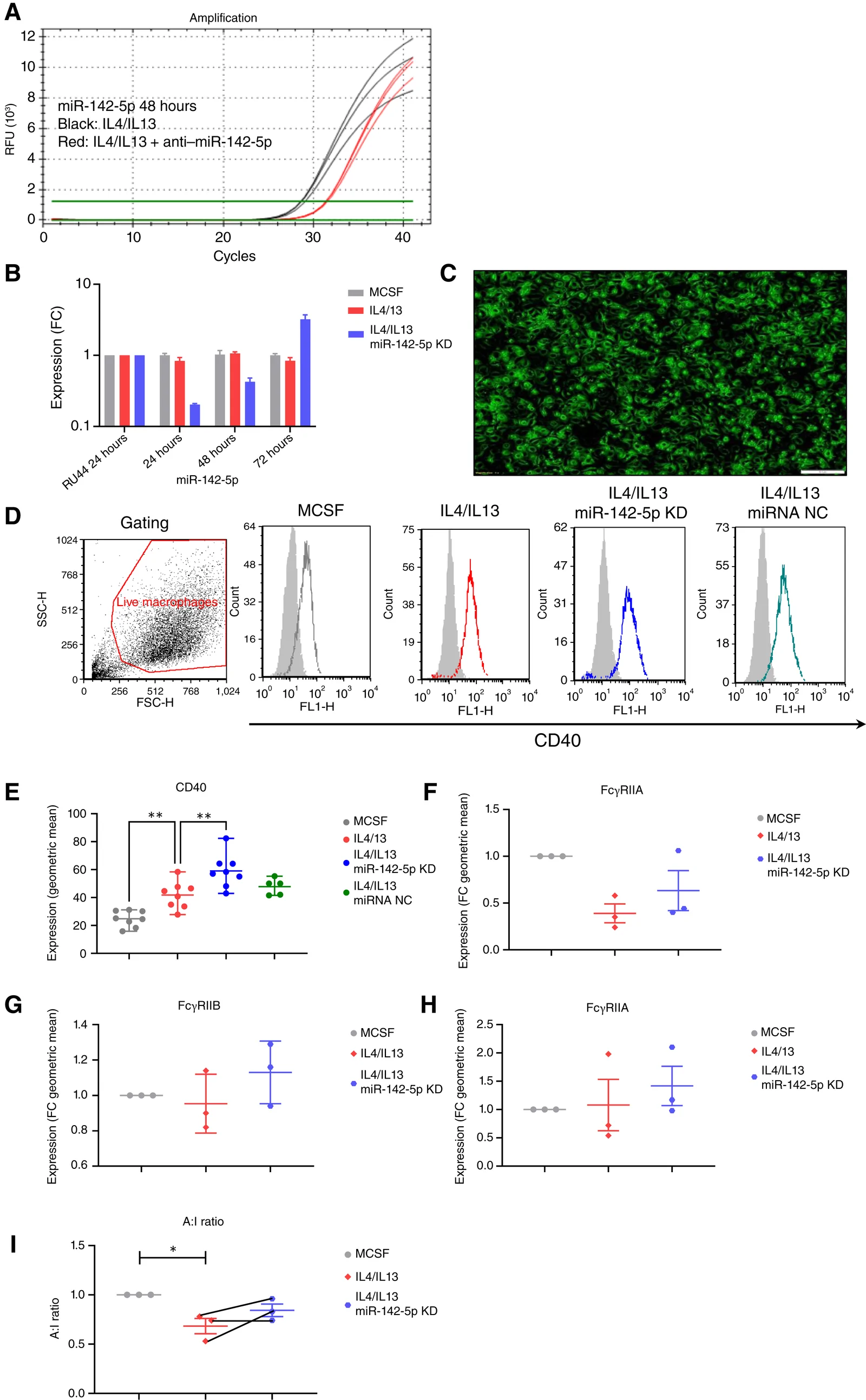
Validation of miR-142-5p KD and downstream analysis of surface marker expression using flow cytometry. KD of miR-142-5p. **A,** qPCR cT curves for transfected IL4/IL13-polarized hMDMs at 48 hours. Cells were polarized in parallel with transfection of an negative control (NC) oligonucleotide or miR-142-5p inhibitor (black and red lines, respectively); technical replicates (triplicates; *n* = 1). **B,** Comparative miR-142-5p expression in MCSF, IL4/IL13, and IL4/IL13 macrophages transfected with miR-142-5p inhibitor at 24, 48, and 72 hours. Data were normalized to RU44 and presented as FC relative to MCSF; the graph shows the mean ± SD (*n* = 3 individual donors). **C,** Morphology of LPS/IFNγ hMDMs 48 hours following polarization and transfection with fluorescein amidites dye (day 9). Images taken using the Olympus CDX41 microscope. **D,** Representative FACS plots showing gating strategy and CD40 expression on nonpolarized (gray open histogram), nontransfected IL4/IL13-polarized hMDMs (red open histogram) and IL4/IL13-polarized hMDMs transfected with miR-142-5p inhibitor or NC oligonucleotide (blue and green open histograms, respectively). CD40 (**D**), FcγRIIA (**E**), FcγRIIB (**F**), and FcγRIIIA (**G**) expression on the above-described hMDM treatment groups after 48 hours of culture. Each graph depicts the mean ± SD FC in expression (geometric mean fluorescence intensity) of the respective molecules for each condition. Horizontal bars indicate the mean and error bars the SD (*n* = 3). Statistical analysis was carried out using a one-way ANOVA test; *, *P* < 0.05; **, *P* < 0.01. **H,** Impact of miR-142-5p KD on FcγR expression. **I,** The graph depicts the A:I ratio (calculated as described in “Materials and Methods”) for the different hMDM treatment groups, presented as FC relative to untreated hMDMs; bars indicate the mean and error bars the SD (*n* = 3). Statistical analysis was carried out using a one-way ANOVA; *, *P* < 0.05; **, *P* < 0.01. FL1-H, fluoresent detector channel height; FSC-H, forward scatter height; RFU, reletive fluroesence units.

To investigate the impact of miR-142-5p knockdown (KD) on macrophage biology, surface receptors previously shown to be highly expressed after inflammatory or suppressive stimulation were assessed on IL4/IL13-treated macrophages transfected with anti–miR-142-5p. Successful transfection of the anti-miRNA into the hMDMs was confirmed using fluorescence microscopy with a FITC-labeled scrambled anti-miRNA control, and KD of miRNA 142-5p was validated by qPCR over time (Fig. 3A–C; Supplementary Fig. S2). We then sought to characterize phenotype by flow cytometry. Notably, the activating receptor, CD40, was significantly upregulated on the surface of IL4/IL13-treated macrophages following anti–miR-142-5p transfection compared with IL4/IL13-treated controls, 48 hours after transfection and polarization (Fig. 3D and E). We also observed changes in FcγR expression in transfected macrophages translating into an overall increase in their FcγR activatory:inhibitory (A:I) ratio (Fig. 3F–I). Given that an increased A:I ratio has been associated with enhanced phagocytic activity in previous studies, we proceeded to assess functional changes in antibody-mediated phagocytosis (10).

### miR-142-5p KD enhances ADCP in immunosuppressive macrophages

**Figure 4.**
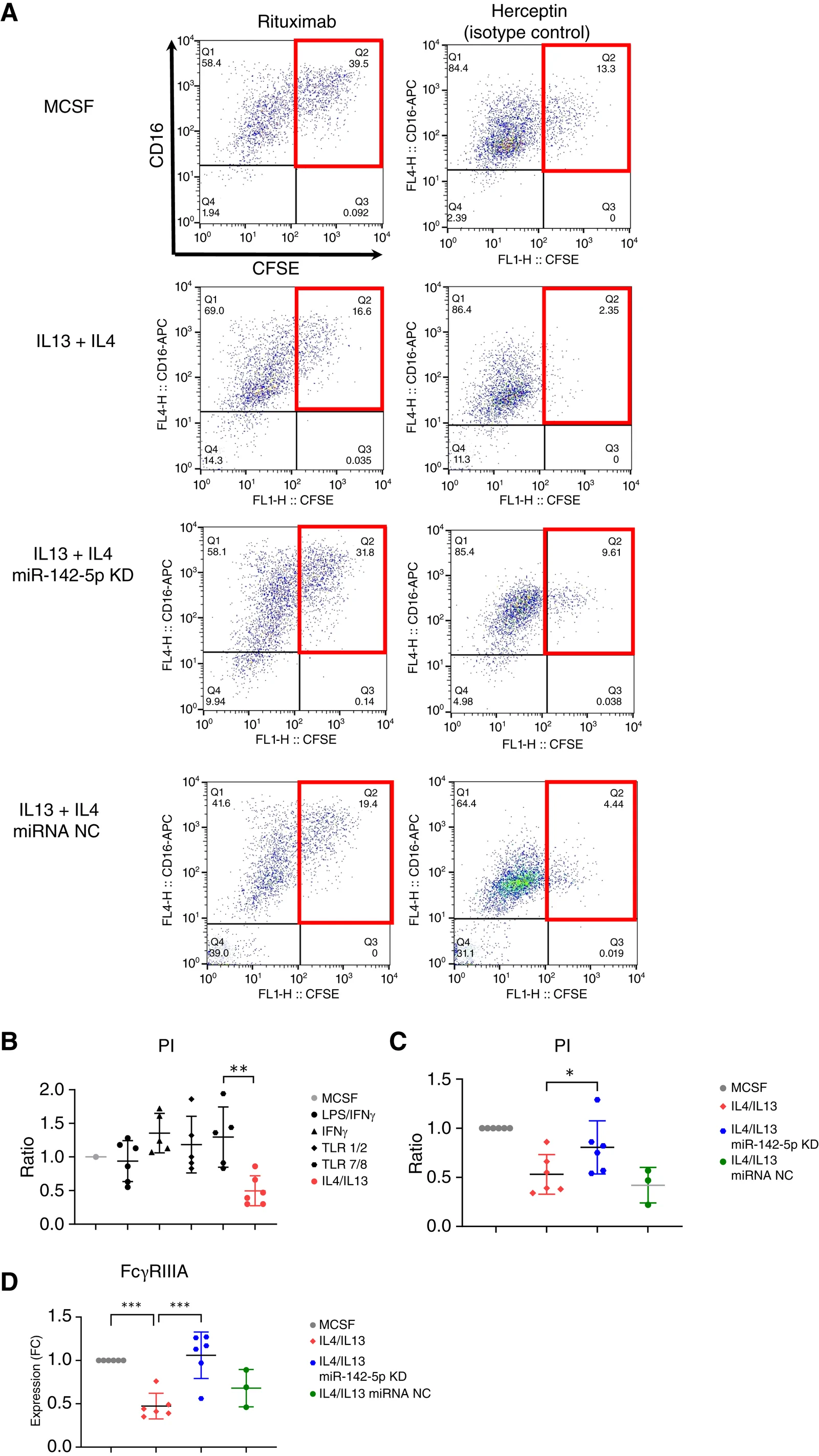
Functional impact of miR-142-5p KD in differentially polarized MDMs. The impact of miR-142-5p KD on polarized hMDM function was assessed in terms of ADCP. **A,** Representative pseudocolor plots showing live gating strategy and plots of CD16 APC versus CFSE for MDMs, differentially polarized in the absence or presence of miR-142-5p inhibitor or negative control (NC) oligonucleotide and cocultured with either for rituximab- or Herceptin-opsonized CLL targets. The proportion of CD16+ CFSE+ cells (top right quadrant, highlighted in red) equates to the degree of ADCP for each condition. **B,** The graph depicts the PI for polarized hMDM populations, calculated as FC in ADCP relative to nonpolarized hMDMs. Bars represent the mean and error bars the SD for each condition; each filled shape represents an individual donor. Between-group comparisons were carried out using one-way ANOVA tests; *, *P* < 0.05; **, *P* < 0.01. TLR, Toll-like receptor. **C,** The graph depicts the PI for IL4/IL13-polarized hMDMs nontransfected or transfected with miR-142-5p inhibitor or NC oligonucleotide controls calculated as FC in ADCP relative to nonpolarized MDMs. Bars represent the mean and error bars the SD for each condition; each filled shape represents an individual donor (*n* = 6). Between-group comparisons were carried out using one-way ANOVA tests. **D,** Graph depicting comparative FcγRIIIA expression presented as FC in geometric mean fluorescence intensity relative to nonpolarized hMDMs. Bars represent the mean and error bars the SD for each condition; each filled shape represents an individual donor (*n* = 6). Between-group comparisons were carried out using one-way ANOVA tests; ***, *P* < 0.001. FL1-H, fluorescence 1-height; FL4-H, fluorescence 4-height.

ADCP was assessed using primary human CLL cells as targets in the presence of anti-CD20 mAb (rituximab). Macrophages polarized with IFNγ and Toll-like receptor had higher ADCP activity with the percentage of double-positive (CFSE+ FcγRIIIA+) cells increased compared with those polarized with IL4/IL13, indicating an enhanced phagocytic capability, confirmed with a statistically significant decrease in the PI of IL4/IL13-treated macrophages compared with other conditions in six independent donors (Fig. 4A and B; ref. 22). Macrophages treated with IL4/IL13 and transfected with anti–miR-142-5p showed a far greater percentage of double-positive cells, indicating enhanced phagocytic activity compared with IL4/IL13-treated controls (Fig. 4C). This was confirmed in six independent donors, with a statistically significant increase in PI in anti–miR-142-5p–transfected macrophages. In addition, a significant increase in the geometric mean of the activating FcγRIIIA in IL4/IL13-treated macrophages transfected with anti–miR-142-5p was seen compared with IL4/IL13-treated controls, which corresponded to overall increased phagocytic activity (Fig. 4D). Less than 4% of cells were double-stained, indicating a lack of phagocytosis, in the presence of the nonbinding isotype control Herceptin across all conditions (Fig. 4A). The mean % double-positive cells in both the isotype antibody group (Herceptin) and target antibody group (rituximab) were higher in anti–miR-142-5p macrophages compared with IL4/IL13-treated controls, indicating that both antibody-dependent and -independent mechanisms may be involved in enhancing the phagocytic capability of the macrophages (Fig. 4A and B). Given these *in vitro* findings, we then sought to translate this into clinical observations by investigating the importance of miR-142-5p expression in combination with CD206^+^ TAMs in treatment-naïve patients with DLBCL and correlate this with survival outcomes.

### miR-142-5p and CD206 co-expression in macrophages correlates with inferior PFS in DLBCL

**Figure 5.**
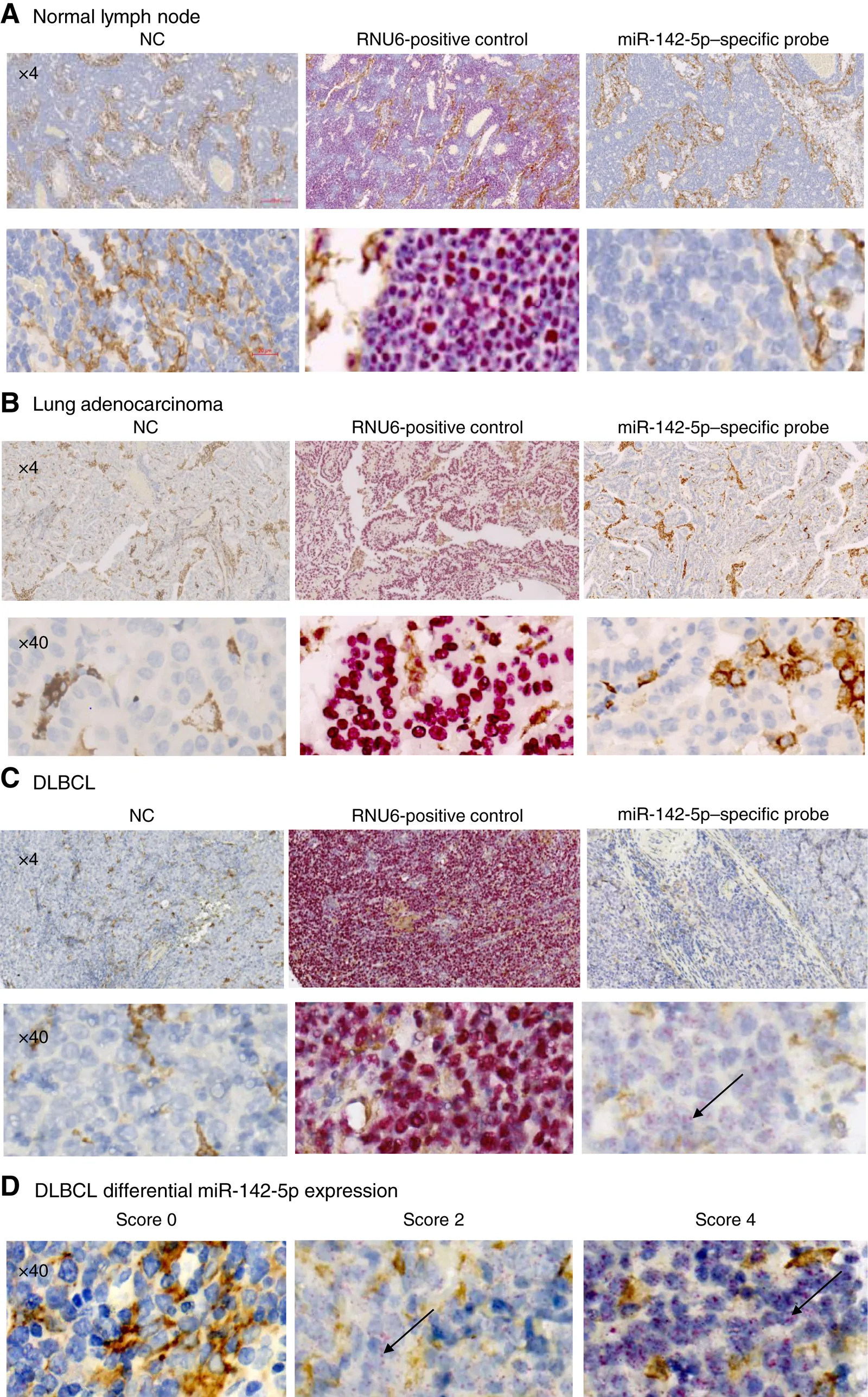
CD206 plus miR-142-5p expression in DLBCL. IHC and RNAScope *in situ* hybridization (ISH) technology were used in combination to assess the expression of CD206 and miR-142-5p, respectively, in FFPE sections obtained from lung adenocarcinoma (**A**), normal lymph node (**B**), and DLBCL samples (**C**). Images were captured using a Z1 upright microscope linked to an AxioCam MRc 5 camera (both ZEISS), following a predefined scanning protocol (blade fold wing stow fold-light 20×), and captured images exported to ImageJ for analysis. Each panel depicts CD206 staining (DAB) combined with an miRNA scrambled probe [negative control (NC), left column], RNU6-specific probe (red, positive control, central column), and miR-142-5p–specific probe (red, right column); magnification, 4× and 40× (top and bottom rows in each panel, respectively). miR-142-5p–specific probe image (40×) in **C** and the score 4 image (40×) in **D** are from the same patient sample.

**Figure 6.**
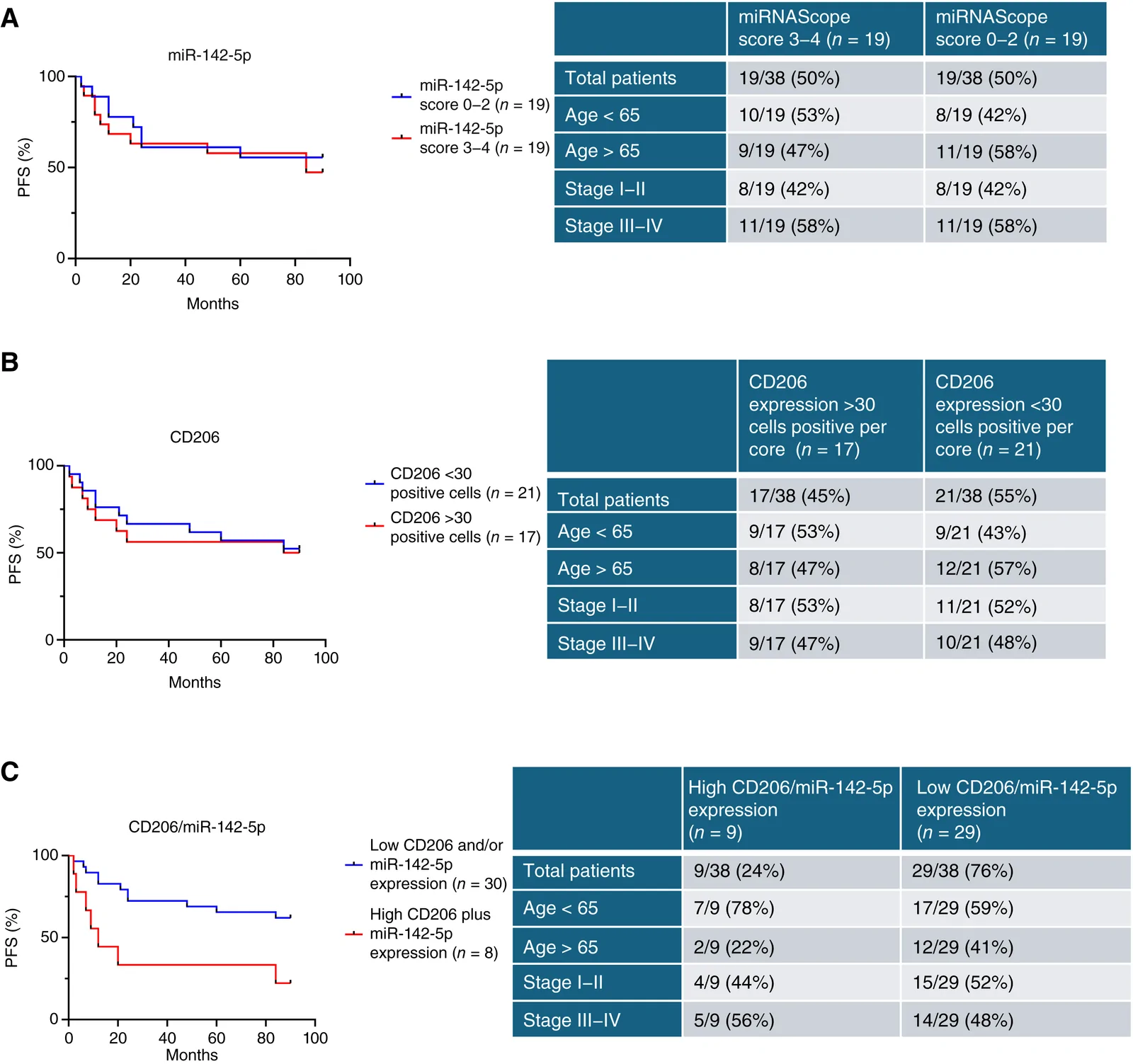
Association among macrophage phenotype, miR-142-5p expression, and survival outcomes in patients with DLBCL. **A,** Kaplan–Meier curves showing (PFS in patients with high (miRNA score of 3–4) versus low (miRNA score of 0–2) miR-142-5p expression (red and blue lines, respectively). **B,** Kaplan–Meier curves showing PFS in patients with high (>30 positive cells) versus low (<30 positive cells) CD206 expression (red and blue lines, respectively). **C,** Kaplan–Meier curves showing PFS in patients with high miR-142-5p and CD206 expression (*n* = 9) compared with those patients with low CD206 and/or miR-142-5p expression; *n* = 38 (median PFS, 12 months vs. not reached; *P* = 0.0096), generated using GraphPad Prism and analyzed using a Gehan–Breslow–Wilcoxon test.

Previous studies have shown that miRNA expression profiles, as determined by bulk RNA-seq, are associated with prognostic outcomes in DLBCL (23, 24). However, bulk analysis does not distinguish the cellular source of miRNA expression, with many miRNAs likely reflecting dysregulation within tumor cells themselves (25). To better evaluate the impact of the miR-142-5p, we sought to investigate miRNA expression in a cell-specific manner in FFPE tissues, i.e., evaluating the expression of miR-142-5p in CD206^+^ macrophages and tumor cells in diagnostic DLBCL samples prior to treatment with rituximab-containing regimes. miRNA scoring was based on a system developed by the manufacturer, with a score of 3 to 4 denoting high expression and 0 to 2 denoting low expression. We then correlated the clinical effect (measured as PFS) with the expression of CD206 and/or miR-142-5p. We also recorded pathologic and clinical data including age, stage of disease, cell of origin, and MUM1, BCL2, and BCL6 status (Supplementary Tables S3–S7). With respect to tissue/tumor-specific staining, there was a lack of miR-142-5p expression in both normal lymph node and lung adenocarcinoma tissues despite a high number of macrophages positive for CD206 staining (Fig. 5A and B). The expression of both miR-142-5p and CD206 was specific to each sample in DLBCL; however, the expression of miR-142-5p was present in both tumor cells and macrophages (Fig. 5C and D). Preliminary analysis revealed that high expression of miR-142-5p alone was not associated with reduced PFS when compared with low expression; similarly, the expression of CD206 (high vs. low) alone was not associated with differences in PFS (Fig. 6A and B). However, high expression of both CD206 and miR-142-5p was associated with significantly inferior PFS (median PFS, 12 months vs. not reached; *P* = 0.0096; Fig. 6C). Given the increase in miR-142-5p observed in macrophages stimulated with IL4/IL13 *in vitro*, its expression may be specific to certain B-cell lymphomas, in which an IL4/IL13 signature is present within the TME.

## Discussion

The B-cell lymphoma TME is considered immunosuppressive, particularly with regard to antibody effector functions, due to elevated expression of the inhibitory FcγRIIB and decreased expression of activating FcγRs (10, 26). Finding ways to tip the balance toward a higher FcγR A:I ratio may therefore be beneficial when treating with direct targeting mAbs such as rituximab, which are central to most treatment regimens (27–32). In B-cell lymphoma, these changes seem to be more important in the macrophage population, with monocytes and neutrophils being relatively unaffected, at least with respect to preclinical models and FcγRs (10). Therefore, reskewing of macrophages toward a more inflammatory phenotype is highly desirable for maximal therapeutic activity of antibody therapies (13, 33).

In our studies, there was an increase in miR-142-5p 48 hours after stimulation with immunosuppressive cytokines reflective of those within the DLBCL TME. In IL4/IL13-treated macrophages, inhibition of miR-142-5p resulted in an increased expression of the activating FcγRIIIA and CD40 compared with nontransfected IL4/IL13-treated macrophages, resulting in a higher FcγR A:I ratio that translated into enhanced ADCP. This inhibition of miRNA was maximal at 24 hours, with changes in downstream surface marker expression seen at 48 hours (Supplementary Fig. S2). Previous studies have shown that pulmonary macrophages expressed high levels of miR-142-5p following exposure to IL4 in a murine allergic asthma model (19). These macrophages repressed the expression of SOCS1, leading to an increased production of anti-inflammatory cytokines CCL17 and TGFβ1 via the JAK2/STAT6 pathway. Transfection of an miR-142-5p inhibitor into these macrophages isolated from murine hepatic and pulmonary tissues suppressed fibroblast activation, reduced the expression of CD206, and reversed the fibrotic changes caused by bleomycin, improving survival outcomes (19, 20). The role of miR-142-5p in macrophages within the TME could therefore be important in maintaining an immunosuppressive state which could be targeted to reverse immunotherapy resistance.

The use of *in vitro* models to replicate an immunosuppressive TME, for example, by analyzing macrophages generated from PBMCs polarized to an immunosuppressive phenotype, is simplistic and does not accurately reflect the complex interactions between cancer cells and different types of immune cells in the context of a tissue. We therefore sought to translate our findings by investigating the expression of miR-142-5p in primary human tumor samples. We found that dual high expression of miR-142-5p and CD206 correlated with inferior PFS in patients with DLBCL who were subsequently treated with rituximab-containing regimes in this preliminary dataset. The expression of miR-142-5p in DLBCL was present in both tumor cells and macrophages that were CD206^+^ in this study and therefore could be specific to the presence of an IL4/IL13 signature within the TME in B-cell lymphomas (34). Cross-talk between tumor cells and macrophages using secreted factors such as cytokines and exosomal miRNAs may be a way for the tumor to maintain immunosuppression (35). We hypothesize that exosomal transfer of miR-142-5p from tumor cells to macrophages within the TME may downregulate the expression of key target genes resulting in a switch to a more immunosuppressive phenotype including upregulation of CD206, coincident with reduced ADCP, and poorer responses to rituximab. Further studies are needed to ascertain the role of exosomal miRNAs within the TME and their potential role as biomarkers in predicting response to treatment (36).

Limitations of this study include the use of single/dual cytokine stimuli to polarize macrophages *in vitro* that are not reflective of the complex immunosuppressive TME. Initially, we studied macrophages stimulated with IL13/IL4 in the context of miRNA expression as it represents a well-defined immunosuppressive polarization status at one extreme of the macrophage activation spectrum (37). After confirming miR-142-5p upregulation in this macrophage population, we then demonstrated the same trend in IL10- and TGFβ-stimulated macrophages. Inhibition of miR-142-5p was maximal at 24 hours, but this inhibition was reversed at 72 hours, which highlights the lack of durability with miRNA when translating this concept into therapeutic drug development. Within the TME, the use of CD206 as a single marker to depict macrophage function in IHC studies was another limiting factor in our analysis when assessing miRNA expression in samples of patients with DLBCL. We chose to use CD206 instead of CD163 in our IHC analysis, as CD163 was downregulated in immunosuppressive macrophages stimulated with IL4/IL13 compared with nonpolarized controls in our *in vitro* work despite it being informative in our initial bioinformatics work.

This study supports the importance of miRNA expression in macrophage activity in B-cell lymphoma and the feasibility of their modulation using anti-miRNA reagents in a cell-specific manner to change phenotype and function (38). Targeting cells of the innate immune system alongside T-cell therapies could improve survival outcomes for patients who suffer treatment resistance or relapse of their cancer following initial therapy. The difficulty of targeting miRNAs in this way needs to be overcome for the potential of miRNA therapeutics to be realized and translated effectively into the oncology clinic (39–42).

## Supplementary Material

Figure S2 Kinetics of miRNA 142-5p expression in different macrophage populations

Table S1 MiRNAs with highest correlation with the immunosuppressive macrophage module (Cyan)

Table S2 Target Scan online network search for miRNAs broadly conserved across vertebrates and cross referenced with known M2-like markers.

Table S3 Baseline characteristics of patients with high (miRNA score of 3-4) versus low (miRNA score of 0-2) miRNA 142-5p expression

Table S4 Baseline characteristics of patients with high CD206 expression (>30 positive cells) versus low CD206 expression (<30 positive)

Table S5 Baseline characteristics of patients with high miRNA 142-5p and CD206 expression compared to those patients with low CD206 and/or miR-142-5p expression

Table S6 Clinical and pathological features of treatment naïve DLBCL FFPE patient samples with low miRNA/CD206 expression.

Table S7 Clinical and pathological details of treatment naïve DLBCL FFPE patient samples with high miRNA 142-5p plus CD206 expression

Figure S1 Comparative phenotype and miRNA expression in macrophages polarised with IL10, TGFbeta, IL4/IL13 verses non-polarised macrophages

## Data Availability

The datasets used and/or analyzed are available from the corresponding author upon reasonable request.
